# Editorial: Effects of plant-microbiome interactions on phyto- and bio-remediation capacity, volume II

**DOI:** 10.3389/fpls.2023.1248248

**Published:** 2023-07-21

**Authors:** Angela Cicatelli, Francesco Guarino, Nuria Ferrol, Piotr Rozpądek, Stefano Castiglione

**Affiliations:** ^1^ Department of Chemistry and Biology “A. Zambelli”, University of Salerno, Fisciano, Salerno, Italy; ^2^ Department of Soil Microbiology and Symbiotic Systems, Spanish National Research Council, Granada, Spain; ^3^ Plant-Microorganism Interactions Group, Małopolska Center of Biotechnology Jagiellonian, University in Kraków, Kraków, Poland

**Keywords:** plant-microbe interactions, abiotic stress, organic and inorganic pollutants, bioremediation, phytoremediation

The plant, with its extensive network of closely associated and mutually interacting microorganisms, can be regarded as a holobiont (Zilber-Rosenberg and Rosenberg, 2008). The interactions within the holobiont rely on a tightly controlled cross-talk between the interacting parties. The plant releases root exudates into the rhizosphere, which nourish microbes and shape plant and soil microbial communities. Potent signalling molecules such as flavonoids and strigolactones play important roles in this interaction process. The composition of the exudates is defined by the plant genotype, developmental stage, exposition to stresses, etc. On the other hand, microorganisms provide fitness advantages to the host plant, including nutrient uptake, growth promotion and protection against abiotic and biotic stress. Detoxification of xenobiotics/pollutants is an important aspect of this protection. Microorganisms affect pollutant mobility and/or their bioavailability, promote their extraction or degradation and, therefore, influence the efficiency and rate of phyto- and bio-remediation.

The holobiont has to be considered as a functional whole, where two essential closely integrated components (genomes) can be recognized. This vision opens new fields of investigation, in terms of biotechnological applications and especially for phyto- and bio-remediation. The current Frontiers in Plant Science Research Topic includes six articles, which shed light on how the holobiont constituents (plant and microbiomes) respond each other to various environmental stresses (drought, salt, climate change and soil pollution) and even to agricultural practices (such as soil amendment).

The beginning of the 21^st^ century is marked by an increase in human population and environmental pollution, coupled with the scarcity of water resources, increased salinization of soils, and a reduction in land available for agricultural purposes.

The effects of climate change and anthropogenic pressure on agriculture require taking measures necessary to secure sufficient crop production. An interesting strategy is the utilisation of microbes from microbiomes either from native sites or contaminated habitats to improve crop production. This can favour plant performance under stress, including drought, salt and heavy metals and be an alternative for chemical fertilizer in sustainable agriculture.

Ganesh et al., focused their research on *Ceanothus velutinus* Dougl. ex Hook (commonly known as snowbrush ceanothus). The isolation of rhizobacteria and subsequent characterization of PGPR features revealed that *C. velutinus* can be an excellent resource for plant growth promoting microbes. This study confirmed that some microbes, associated to native plants, induce local or systemic stress mitigation response mechanisms that facilitate vegetation under drought stress.

Understanding the physiological, metabolic, and biochemical responses of plants and their associated microbial communities to salt stress will be extremely important to select and cultivate salt tolerant plant species or crops. Pereira et al. dissected the response of *Festuca rubra* subsp. *pruinosa*, with an engineered mycobiome to salinity and nutrient deficiency. The Authors showed that inoculation of plants with selected fungal endophytes improved the performance of plants inhabiting the saline habitat of sea cliffs. The beneficial action of the treatment most probably resulted from fine-tuning of plant Na^+^/K^+^ homeostasis. Most importantly though, the Authors explored the role of individual microorganisms in plant adaptation to salinity. Soil is a limited and fragile resource, that is often affected by unsustainable human activity such as overuse of agrochemicals, etc. Due to its high efficiency and stable performance, the herbicide 4-Chloro-2-methylphenoxyacetic acid (MCPA) is commonly used, mainly for intensive agriculture. However, MCPA is highly toxic to aquatic- and soil-inhabiting organisms. To accelerate MCPA soil removal and to prevent its dispersal in the environment, a phytoremediation strategy has been proposed. This strategy relies on amending the soil with syringic acid (SA), a secondary metabolite synthesized by cucurbits. This compound was shown to act as an herbicide and is also able to shape plant associated bacterial communities. In the article by Mierzejewska et al., bacterial communities of plants and soil treated with SA was assessed. The Authors showed that soil amendment with SA and cultivation of zucchini resulted in enhanced removal of MCPA. Improved MCPA removal was also associated with an increased bacterial diversity, altered microorganism community structure and with an increased number of bacterial genes responsible for MCPA degradation.

Facilitation of nutrient uptake and metal stress protection are paramount features of the plant and rhizosphere microbiota. Fan et al. investigated the rhizosphere microbiome assembly process in relation to plant metal tolerance and its role in plant adaptation to Cd toxicity using *Sedum alfredii* ecotypes with different Cd tolerance and accumulation and high throughput metagenomics. This study provided insights into the role of rhizosphere microbiome in the biogeochemical nutrients and metals cycles that can affect plant fitness in polluted soils.

Plant-fungal interactions have been extensively studied in the context of application of arbuscular mycorrhizal fungi (AMF) in phytoremediation for reclamation of metal polluted soils. AMF are ubiquitous soil-borne microorganisms with a notorious contribution to plant health. They establish mutualistic symbioses with most land plants, improving their mineral nutrition and tolerance to biotic and abiotic stresses. In their study, Jia et al. evaluated the effects of four different AM fungal species on the eco-physiological responses of *Imperata cylindrica* L. plants, grown under differing soil nitrogen conditions (low and high content), with the aim to identify the most efficient AM fungal species to improve *I. cylindrica* resistance to copper tailings. This study provided insights to be utilized for eco-friendly restoration of metal polluted sites, mainly characterized by nutrient-poor soil conditions.

Improving the efficiency of plant bio-stimulation by microorganisms with organic and inorganic soil amendments has been an interesting and highly effective practice in phytoremediation strategies. The report by Eltahawy et al. showed that the application of well-known plant growth promoting microorganisms in combination with moringa extracts and Silicon-NanoParticles (SiNPs), can improve plant performance and metal uptake of spinach cultured under metal toxicity. The Authors clearly showed that the integrative approach yielded the best results.

## Author contributions

AC: Writing - Original Draft. NF, PR, FG, SC: Writing - Review & Editing. All authors contributed to the article and approved the submitted version.

## References

[B1] Zilber-Rosenberg I. Rosenberg E. (2008). Role of microorganisms in the evolution of animals and plants: the hologenome theory of evolution. FEMS Microbiol. Rev. 32 (5), 723–735. doi: 10.1111/j.1574-6976.2008.00123.x 18549407

